# School as ideal setting to promote health and wellbeing among young people

**DOI:** 10.34172/hpp.2020.50

**Published:** 2020-11-07

**Authors:** Manuela Pulimeno, Prisco Piscitelli, Salvatore Colazzo, Annamaria Colao, Alessandro Miani

**Affiliations:** ^1^UNESCO Chair on Health Education and Sustainable Development, Naples, Italy; ^2^Doctorate in Human Relations Sciences, University of Bari “Aldo Moro”, Bari, Italy; ^3^Euro Mediterranean Scientific Biomedical Institute, ISBEM, Bruxelles, Italy; ^4^Department of History, Society and Human Studies, University of Salento, Lecce, Italy; ^5^Department of Clinical Medicine and Surgery, Federico II University School of Medicine, Naples, Italy; ^6^Department of Environmental Science and Policy, University of Milan, Milan, Italy; ^7^Italian Society of Environmental Medicine, SIMA, Milan, Italy

**Keywords:** School, Students, Prevention, Health, Education

## Abstract

**Background:** Nowadays, young people face several health challenges. As children and teenagers spend most of their time in the classroom, schools may have the opportunity to positively influence students’ quality of life, playing a crucial role in fostering their health. The aim of this review was to analyze evidence that demonstrated why school is the ideal setting for thepromotion of young generations’ wellbeing.

**Methods:** We have reviewed the available literature about health promotion in school setting, searching for articles and books published from 1977 to 2020. A total of 74 articles and 17books were selected and assessed.

**Results:** The promotion of students’ wellbeing could reduce the prevalence of measurable unhealthy outcomes and improve their academic achievements. At least 80% of all cases of heart diseases, strokes, type 2 diabetes and one third of all cancers can be prevented through health education. In this perspective, primary prevention and health promotion should start as early as possible, finding in the school the ideal setting of action. Effective school-based preventive approaches should raise students’ motivation towards a personal interiorization of health knowledge and develop in young people a critical thinking about harmful consequences of the most common risky behaviours. Educators should receive adequate training concerning health topics and become expert in the most innovative approaches to effectively engage students in adopting healthy lifestyles.

**Conclusion:** As primary educational institution, school should integrate students’ health promotion in its ordinary teaching and learning practices in the perspective of "better health through better schools".

## Introduction

In the 21st century, young people face several health challenges. On one hand, a high intake of total fat, free sugars, and salt, along with the lack of physical activity, have contributed to increase chidren’s obesity at alarming rates; on the other hand, adolescents’ lives are threatened by addictive and risky behaviours (e.g. tobacco smoking, alcohol, substance abuse, unprotected sex, inter-personal violence, intentional self-harm, extreme “deadly selfies”).^1^

**Figure 1:**
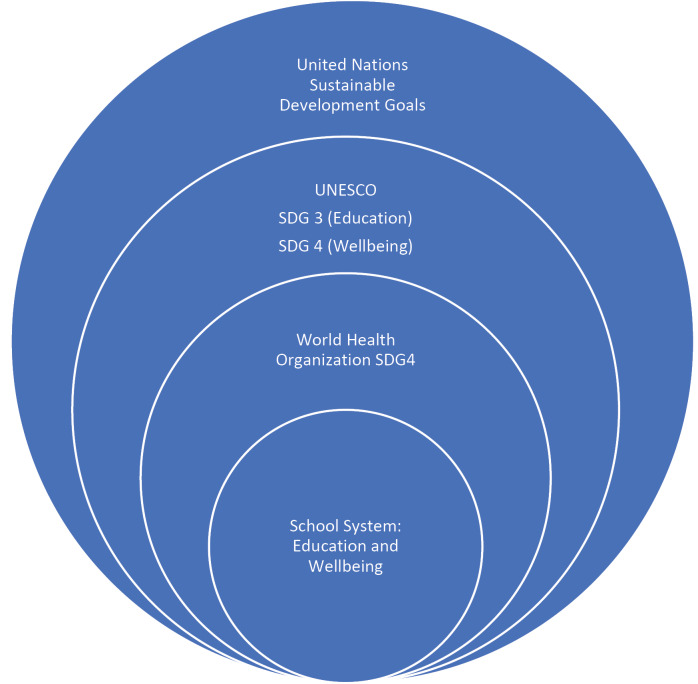
School System: education and wellbeing promotion in the frame of Sustainable Development Goals (SDGs) set by the United Nations

In this context, the United Nations (UN) recognize education as essential for children’s global growth and key factor for improving young people health. The UN agency UNESCO is focused on turning into actions the educational commitments set by the United Nations “2030 Agenda for Sustainable Development”,^2^ while our Unesco Chair on Educational Health and Sustainable Development is specifically aimed at addressing the Sustainable Development Goals (SDGs), paying particular attention to the SDG3 concerning the promotion of “good health and wellbeing” and SDG4 to “ensure inclusive and equitable quality education and promote lifelong learning opportunities for all” (Figure 1). The school-based activities of our Unesco Chair are targeted to the prevention of harmful habits and promotion of wellbeing among schoolchildren living in Southern Italy, an area characterized by a widespread presence of vulnerable social groups at higher risk of developing unhealthy behaviours, due to socio-economic disadvantages.^3^

In the view of the holistic individual development, the primary commitment of school systems – along with students’ academic achievements – should be the improvement of children’s physical, mental and social wellbeing.^4^ In our vision, school may represent the optimal setting to display educational health-related interventions,^5-8^ as educators can have the opportunity to positively influence – day by day – students’ life-long learning and work to reduce health inequalities among young people.^3^ Our first systematic review examined a number of studies concerning the effectiveness of multicomponent narrative-based strategies to improve healthy eating habits and decrease risk factors for overweight and obesity in schoolchildren.^9^ More broadly, beyond the specific topic of healthy nutrition, children’s global wellbeing might be systematically promoted at school by adopting innovative active approaches, where young people are not considered passive or simple audience, but are engaged in practical actions about healthy lifestyles (i.e. balanced nutrition and physical exercise, no smoking, no alcohol, no drugs etc).^10,11^

The World Health Organization (WHO) suggests that health literacy should be incorporated in the core curriculum as children enter school, supported by a health-promoting school environment.^12-14^ A comprehensive school commitment towards students’ global wellbeing is expected to positively impact both children’s behaviours and their families.^15^ For that reason, we question why scholastic institutions are not widely and systematically engaged in a proper path – according to specific keypoints set by WHO – to become “health-promoting schools” (Table 1), able to prevent students’ risky behaviours.^16,17^ This review is specifically aimed at providing evidence-based justification to consider school as ideal setting for the promotion of young generations’ wellbeing.

## Materials and Methods

### Information sources, search strategies and study selection

In this short narrative review, we have explored the available literature concerning the rationale for promoting children’s wellbeing in school setting. The review has been carried out by PhD candidates and academic experts in Human Sciences together with Medical Doctors according to the main items reported in the PRISMA checklist 2009.^18^ We searched on Web of Science and Google Scholar for original articles and books published from 1977 to 2020 by using a search strategy based on the following keywords: “health promotion” OR “primary prevention” OR “wellbeing” AND “students” OR “school”. Data extraction was performed by a PhD candidate and separately confirmed by a medical doctor. Additionally, we used citation tracking to detect other papers concerning health promotion in school setting. Exploration of heterogeneity of the studies was performed by assessing their quality (i.e. level of evidence). Interpretation of the findings has been conducted in the frame of current knowledge.^19^

### Exclusion criteria

We have excluded studies concerning psychopathology, psychiatric disorders, drug/alcohol addiction or eating disorders, and therapeutical applications. We have also left out articles regarding clinical topics such as autism spectrum conditions, specific learning difficulties, cognitive or sensory/physical deficits. Moreover, we removed all the articles presented in language other than English and Italian.

### Synthesis of search results and summary measures

A total of 74 articles and 17 books’ chapters were selected for the review. We have briefly summarized definitions of health, healthy lifestyles, health promotion, primary prevention, protective and risk factors, considering wellbeing (in its three dimensions of physical, emotional/mental and social health) as the main goal of every educational practice, and school system as the ideal setting to perform educational health-related interventions.

## Results

### Students’ wellbeing promotion and academic achievements: a virtuous circle

Since education and wellbeing are intertwined dimensions, an important “mission” of any educational system is to ensure that students are healthy and able to learn.^1^ Children spend most of their lifetime in classroom and that’s why school can be the natural setting for promoting their health. By working everyday with pupils, teachers have a crucial role in positively influencing their global development and equipping them with the knowledge, attitudes, and skills needed to protect and maintain their healthy habits for the entire life.^20,21^

In the socio-cognitive perspective, school should educate young people to take responsibility for their own health since the early childhood.^22^ A correct approach towards health in daily life encourages the development of children’s self-efficacy, which represents the ability to maintain healthy lifestyles during the life and enjoy the benefits of behavioural changes acquired. This emerging interest towards students’ positive dimensions (such as self-esteem, happiness and resilience) should represent a new priority for school staff and families, to be addressed in a synergic effort.^23,24^

**Figure 2:**
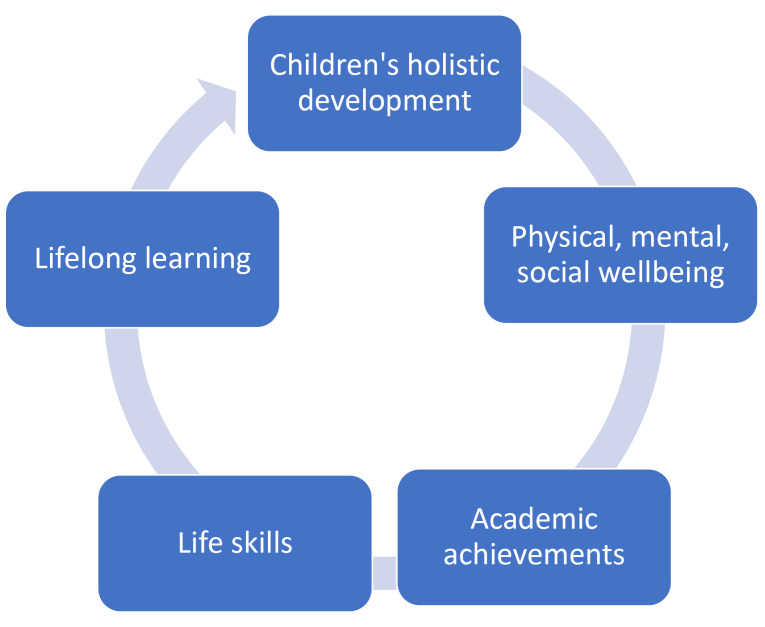
Educational system goals

It is clear that school system is a strategic social environment that can impact children’s wellbeing, although in the last decades school has mainly focused on cognitive and academic achievements rather than adopting a comprehensive children’s care model.^4^ However, as documented in various studies, the wellbeing of the students has also an undoubtable impact on their learning outcomes and should be considered by teachers as a crucial dimension to work on.^25^ Therefore, health promotion can’t remain a marginal aspect of teacher work, as it has the potential to create a ‘virtuous circle’ that makes students able to reach better academic attainments and to improve health outcomes (Figure 2).^26^ Children with social and emotional problems usually show negative results at school, but at the same time those pupils who are experiencing academic difficulties might present increased social and emotional complications.^27,28^ On the other hand, children who perform well at school seem to enjoy better health and have access to more opportunities during their lives.^29^

WHO has started in 2014 a specific “Health Promoting Schools framework” (HPS) to integrate health educational goals in a holistic perspective at school. This programme has shown to positively influence students’ behaviours at least for those interventions having the following endpoints: body mass index, physical activity, fruit and vegetables consumption, prevention of tobacco use and being bullied.^30^ Despite this evidence regarding the potential benefits of school-based health interventions, nationwide structured and well planned health promotion strategies are still lacking. To achieve this goal, health-related contents may be embedded in the school curricula as core discipline, or could be integrated in a health-carrier discipline such as science, or even delivered as extracurricular programme.^14^

The complexity of nowadays requires a deep change in teaching and learning practices, shifting the focus from the mere transmission of notions to active and motivational approaches, able to equip students with a fruitful knowledge and a wide range of life skills. This aspect is also relevant in the field of health education: teachers need to master an array of participatory activities such as class discussions, debates, case analysis, brainstorming, small working groups, peer teaching, co-writing, co-creating projects, educational games and simulations, storytelling, audio and visual laboratories (e.g. arts, music, theatre, dance etc.), in order to enhance students’ health learning outcomes.^31,32^

Moreover, the accomplishment of multifaceted and authentic tasks over a long period of time, along with providing opportunities to reflect on the health-based learning experiences from different points of view, allow students to acquire those transversal skills they need in the real life. These innovative approaches are helpful in involving pupils in the control of the learning environment^33,34^ and can be also useful to generate a respectful climate in the classroom, where pupils can freely practice social skills and lower anxiety due to competition or pressure of success.^35^ Furthermore, researches on anti-bullying programmes have proved that structures, conditions, and learning settings (school environment) are at least as significant as individual factors.^36,37^ Finally, school-based health promotion is more successful if a “whole-school approach” (based on comprehensive school policies) is adopted, paying also attention to school physical environment (appeal and sustainability of buildings, grounds and surroundings). Community links are an additional relevant dimension, because working together with families or communities (in collaboration with available health professionals) help schools in more effectively spreading a “culture of prevention”.^38^

### Primary prevention and education: a scientific justification for school-based interventions

According to the World Health Organization, health is a human right defined as “a state of complete physical, mental and social wellbeing and not merely the absence of disease or infirmity”^39^ and itis influenced by culture, which plays an important role in shaping quality of life perception, both for individuals and communities. Thus, health can be considered as a universal dimension of human culture, reflecting socio-cultural values, traditions, and beliefs shared by a community of people.^40-43^ In light of this wide-ranging concepts of health, also healthy lifestyles could be regarded as complex cultural schemes, involving different aspects such as nutrition, physical activity, work/leisure time, and environmental protection.^44,45^ The efficacy of health education at school can only be evaluated if taking into account multidimensional factors within a comprehensive view of health.

As pointed out by positive psychology, it is fundamental to foster physical, mental/emotional and social wellbeing of individuals since the early childhood, shifting from being focused on diseases prevention to wellbeing promotion, namely from risk factors to protective factors.^46^ Both primary prevention and health promotion approaches are focused on proactively maintaining people healthy, ensuring this change of views.^47^

According to the medical paradigm, three levels of preventive interventions are possible. Primary prevention (universal provision of information about healthy lifestyles) corresponds to health promotion and can be managed at school or community level, while secondary (early diagnosis of risky behaviours in selected population), and tertiary prevention (rehabilitative/dedicated interventions) concern medical field and require professional operators.^48^

The knowledge about protective and risk factors (that belongs to the domain of primary prevention) is useful to plan psycho-socio-pedagogical interventions in school setting that might increase the benefits of protective factors (i.e. resilience, empathy and other soft skills, useful as personal resources or coping strategies to deal more effectively with stressful events).^49-51^ On the other hand, risk factors are described as individual or environmental characteristics that predispose to the early onset of problems (including school dropouts, substance abuse, delinquency, violence, and early pregnancies), usually overlapping in vulnerable social groups.^52^

At the present time, the prevention of emotional problems among young people, leading to possible social deviations, has become one of the most urgent educational emergencies so that primary prevention represents an important educational commitment.^53^ Educational institutions face also the challenge of reducing health inequalities among students and their exsposure to risk factors associated to a higher probability of future problematic behaviours.^54,55^ In particular, school system and teachers are asked to reinforce the points of strength (emotional and social skills) of the students, spreading “a warm blanket of prevention”, instead of adopting a regulatory and stigmatizing style towards already marginalized children or teenagers.^56^ This means encouraging young people to make healthy choices, in order to reduce the risk of developing emotional/social difficulties and future chronic diseases.

**Figure 3:**
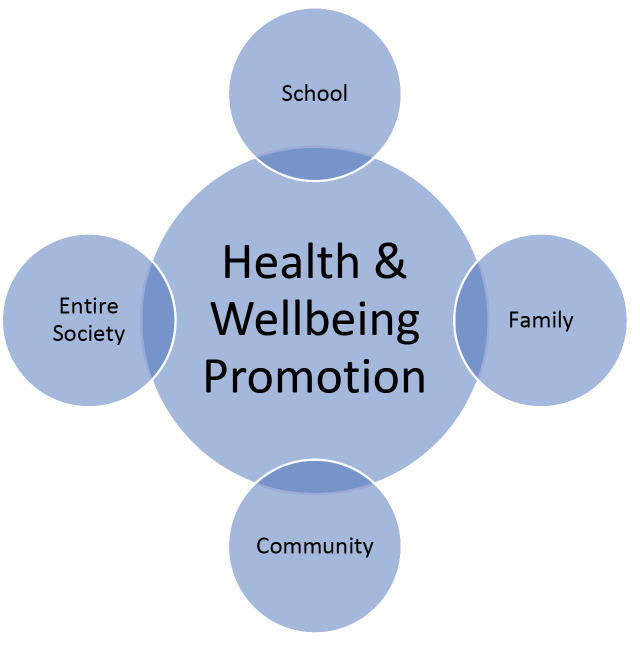
Stakeholders involved in Health and Wellbeing Promotion

Indeed, the World Health Organization has demonstrated that many early deaths are avoidable: at least 80% of all cases of heart diseases, strokes, type 2 diabetes and one third of all cancers can be prevented through health education.^3^ In this perspective, as children’s health is a valuable resource for communities, primary prevention represents a necessary investment for our present and future.^57^ A society that wants to live better should ask each stakeholder to take a piece of responsibility and invest in promoting healthy lifestyles since childhood (Figure 3). Going beyond the mere academic achievements that students are expected to acquire, every educational practice should provide children with the basis for personal self-realization, helping them to grow up as confident learners and responsible citizens for individual and collective health.^58,59^

The attention to students’ wellbeing (physical, social and mental condition) should become part of any pedagogical design that wants to be effective in preventing socio-emotional difficulties and risky behaviours (i.e. addictions to alcohol, tobacco, and drugs). Educators must encourage the adoption of healthy lifestyles and foster the development of critical thinking towards unhealthy behaviours and their physical, psychological and social consequences.^60^

From a pedagogical point of view, the principle of prevention is one of the fundamental concepts of education, in the perspective of life-long learning and people empowerment. Empowered students can be able to trigger processes of social progress in their communities, moving from a passive state to an agency asset and expressing a transformative potential on their communities.^61^

The promotion of children’s health is not only a matter of preventive medicine, but it involves educational and ethical dimensions of social responsibility aimed at increasing young people consciousness and responsibility for their own and other people’s health. Therefore, while working on students’ motivation towards healthy lifestyles, school can raise their awareness about sustainable development topics, as health and environment are strictly interconnected.The adoption of healthy lifestyles – which turns into responsible consumers’ choices (ethical consumption) – is linked to the concepts of ecological, social and economic sustainability, as well as to those of solidarity, peace, equity and legality.^62,63^

Finally, promoting students’ health at school has been found to engage in healthy habits also families and communities (a kind of multiplier effect): children can become health trainers of their parents, relatives and friends, impacting positively the entire society.^15^ Due to its social commitment, school needs the support of all the private and public social actors, in order to overcome the obstacles that arise in the educational path, and build up a comprehensive “preventive system”, able to foster healthy protagonism of the “youngest part of the society”.^64^

## Discussion

Everybody has the right to reach a state of wellbeing in which his or her own talents are fully accomplished, providing a personal contribution to the society.^65^ Since education and health are interrelated, educational system can be considered among the most committed institutions for the promotion of young people’s wellbeing, together with families and communities.^66,67^ However, the changes in social relationships occurred in the last decades (increase in the number of divorces, births outside marriage and family mobility), along with the difficulties due to recent economical crises, make even more crucial the comprehensive educational role of the school.^68-70^

Working for prevention and bringing health information to students represents an intrinsic ethical duty for any scholastic institution, so that teachers – who are already recognized as “promoters of culture” – should become also “health promoters” and “emotional trainers” of their students.^71^ Health education should inform the ordinary teaching activities, becoming part of the daily work of school staff, who have the responsibility to guide students towards the adoption of healthy lifestyles, developing all their cognitive, affective, spiritual and social aspects, especially in a context characterized by an increasing absence of parental support at home.^72-74^

Indeed, effective school-based preventive approaches are those that raise students’ motivation towards healthy habits and foster their critical thinking about harmful consequences of the most common risky behaviours. In this perspective, teachers should boost students’ problem solving and judgment attitudes necessary for protecting their health, working on skills such as communication, assertiveness, self-management, rejection of influences, conflict resolution and negotiation with peers and adults.^75^ The adoption of meaningful contents, methods and tools can ensure a deep and “transformative” learning process, and generates a personal interiorization of knowledge in young people.^76^ Furthermore, a classroom climate of mutual trust and support – where each pupil is an equal participant – encourage students to find by themselves own life projects, following their personal interests and inclinations.^77,78^

The modern educational challenges call for reviewing and updating teaching/learning practices, in order to implement promotional and motivating strategies – with a long-wide-deep learning perspective – thus addressing the limitations of traditional education that does not always satisfy the needs of the new generations.^79,80^ At the same time, invasive or regulatory style should be avoided to reduce the risk of stigmatizing already vulnerable children.^81^ It is possible to overcome the vertical transmission of knowledge based on passive acquisition of information by adopting experiential and participatory approaches such as role playing, debates, tasks of reality, artistic laboratories,^31^ that help students’ to develop transversal competences and personal re-construction of knowledge, stimulating their agency. Active, motivational and participatory teaching/learning methodologies are also useful to set a healthy supportive school environment, where positive values are shared by the students, growing up as socially skilled citizens, able to select and build up their own learning, manage properly their time and apply in real life the knowledge acquired.^82^

Health educational interventions should start as early as possible, addressing all areas of children’s growth (physical, emotional, social and cognitive development)^83^ and should be planned at different levels of operation (with a structured and continuous monitoring of the processes and outcomes): universal programmes for the whole school or targeted preventive actions focused on most vulnerable groups. Health promotion impacts on the whole school population, while preventive interventions are more effective in those groups at higher risk. Health-promoting interventions implemented for disadvantaged children since early stage of life have been proved to be effective in coping with several forms of social marginalization and inequalities.^84^ For this reason, sub-populations of children suffering from socio-emotional problems should be identified in advance (paying special attention to those pupils belonging to socio-economically disadvantaged families), by detecting the presence of ‘warning signs”, such as disturbing behaviours, school refusal, or unusual deviations in their academic profile.^85^

The urgency of putting more efforts on health literacy at school is also triggered by the COVID-19 pandemic and other possible challenges arising from the altered ecosystems balance due to human activities.^86^ Indeed, health promotion is strictly related to education for sustainable development, and the entire school system should deal also with the unavoidable task of environmental protection throughout a systemic strategy. The goal is to stimulate students’ citizenship skills, in particular their sense of responsibility towards personal and collective health, thus empowering young people to take action for a more healthy and sustainable society and to claim – as informed citizens – for policies that positively impact their health and the environment.^87,88^

However, even though there is a strong evidence for implementing health education in school setting, the effects of this kind of interventions are variable and there is no guarantee of success, unless a full commitment of teachers and school staff is displayed. It must be also considered that every organization, including school system, has to deal with the low propensity of teachers to make full use of all the new training opportunities and accept to modify their current educational practices.^89,90^ Moreover, schools have to cope with the lack of financial resources and expert staff (e.g. PhD candidates, professional health services, pedagogical and psychological consultants potentially useful for specific targeted interventions), that could be possibly provided to the school system by a stable cooperation with private and public stakeholders.^91,92^

## Conclusion

Scientific evidence demonstrates that school can be the ideal setting to implement health-related interventions aimed at fostering young people global growth.^93^ Health promotion at school could be effective in improving both students’ wellbeing and their academic achievements, thus generating a virtuos circle. As primary educational institution, school might integrate children’s health promotion in its ordinary teaching and learning practices through a specific revision of the curricula. Educators should be adequately trained on how to raise students’ motivation towards healthy/sustainable lifestyles and display the most innovative participatory methodologies, in order to effectively convey health knowledge to young people, fostering at the same time their critical thinking about harmful consequences of risky behaviours. As Unesco Chair, we highlight that primary prevention should start as early as possible by carrying out well-structured health educational interventions, finding in teachers the most committed social actors, in the perspective of “better health through better schools”.^94^

## Acknowledgments

The UNESCO Chair on Health Education and Sustainable Development and the Italian Society of Environmental Medicine are grateful to the UNESCO Assistant Director-General for Education Dr. Stefania Giannini and her staff.

## Funding

This research has been carried out in the frame of institutional activities of the UNESCO Chair on Health Education and Sustainable Development, without receiving any external funding or economical support.

## Competing interests

All the authors declare that they have no competing interests.

## Ethical approval

Not applicable.

## Authors’ contributions

MP, PP, AM, SC, and AC conceived, wrote and revised this review.

**Table 1:** Keypoints identified by WHO to set up “health promoting schools”

*A health promoting school is one that constantly strengthens its capacity as a healthy setting for living, learning and working*
**A health promoting school:**
Fosters health and learning with all the measures at its disposal.
• Engages health and education officials, teachers, teachers' unions, students, parents, health providers and community leaders in efforts to make the school a healthy place.
• Strives to provide a healthy environment, school health education, and school health services along with school/community projects and outreach, health promotion programmes for staff, nutrition and food safety programmes, opportunities for physical education and recreation, and programmes for counselling, social support and mental health promotion.
• Implements policies and practices that respect an individual's wellbeing and dignity, provide multiple opportunities for success, and acknowledge good efforts and intentions as well as personal achievements.
• Strives to improve the health of school personnel, families and community members as well as pupils; and works with community leaders to help them understand how the community contributes to, or undermines, health and education.
**Health promoting schools focus on:**
• Caring for oneself and others.
• Making healthy decisions and taking control over life's circumstances.
• Creating conditions that are conducive to health (through policies, services, physical/social conditions).
• Building capacities for peace, shelter, education, food, income, a stable ecosystem, equity, social justice, sustainable development.
• Preventing leading causes of death, disease and disability: helminths, tobacco use, HIV/AIDS/STDs, sedentary lifestyle, drugs and alcohol, violence and injuries, unhealthy nutrition.
• Influencing health-related behaviours: knowledge, beliefs, skills, attitudes, values, support.

**Source:**https://www.who.int/school_youth_health/gshi/hps/en/.

## References

[1] The United Nations Development Group. “Together Possible: Gearing up with 2030 Agenda.” Available from: https://undg.org/document/together-possible-gearing-up-for-the-2030-agenda. Accessed 10 March, 2019.

[2] UNESCO. Education 2030: Incheon Declaration and Framework for Action for the Implementation of Sustainable Development Goal 4. Available from: http://uis.unesco.org/sites/default/files/documents/education-2030-incheon-framework-for-action-implementation-of-sdg4-2016-en_2.pdf. Accessed 10 March, 2019.

[3] World Health Organization (WHO). Chronic Diseases and Health Promotion. WHO; 2018. Available from: https://www.who.int/chp/chronic_disease_report/part1/en/index11.html. Accessed 21 March, 2019.

[4] Adams K, Monahan J, Wills R (2015). Losing the whole child? a national survey of primary education training provision for spiritual, moral, social and cultural development. Eur J Teach Educ.

[5] Banerjee R, McLaughlin C, Cotney J, Roberts L, Peereboom C. Promoting Emotional Health, Well-being and Resilience in Primary Schools. Public Policy Institute for Wales, University of Sussex; 2016. Available from: http://ppiw.org.uk/files/2016/02/PPIW-Report-Promoting-Emotional-Health-Well-being-and-Resilience-in-Primary-Schools-Final.pdf. Accessed 21 March, 2019.

[6] Inchley J, Muldoon J, Currie C (2007). Becoming a health promoting school: evaluating the process of effective implementation in Scotland. Health Promot Int.

[7] Stewart-Brown S. What is the Evidence on School Health Promotion in Improving Health Orpreventing Disease and, Specifically, What is the Effectiveness of the Health Promoting Schools Approach? Copenhagen: WHO Regional Office for Europe; 2006.

[8] Young I (2005). Health promotion in schools--a historical perspective. Promot Educ.

[9] Pulimeno M, Piscitelli P, De Marco E, Colazzo S (2018). Narrative-based strategies to promote healthy eating behaviours in childhood: a systematic review. MeTis - Mondi educativi. Temi, indagini, suggestioni.

[10] Auster ER, Wylie KK (2006). Creating active learning in the classroom: a systematic approach. J Manag Educ.

[11] Michel N, Cater III JJ, Varela O (2009). Active versus passive teaching styles: an empirical study of student learning outcomes. Hum Resour Dev Q.

[12] World Health Organization (WHO). School Policy Framework: Implementation of the WHO Global Strategy on Diet, Physical Activity and Health. Geneva, Switzerland: WHO; 2008. Available from: https://www.who.int/dietphysicalactivity/SPF-en-2008.pdf?. Accessed 10 March, 2019.

[13] Paakkari L, Paakkari O (2012). Health literacy as a learning outcome in schools. Health Educ.

[14] Kilgour L, Matthews N, Christian P, Shire J (2015). Health literacy in schools: prioritising health and well-being issues through the curriculum. Sport Educ Soc.

[15] Kolbe LJ (2019). School health as a strategy to improve both public health and education. Annu Rev Public Health.

[16] Smith BJ, Potts-Datema W, Nolte AE (2005). Challenges in teacher preparation for school health education and promotion. Promot Educ.

[17] Jourdan D, Samdal O, Diagne F, Carvalho GS (2008). The future of health promotion in schools goes through the strengthening of teacher training at a global level. Promot Educ.

[18] Moher D, Liberati A, Tetzlaff J, Altman DG (2009). Preferred reporting items for systematic reviews and meta-analyses: the PRISMA statement. PLoS Med.

[19] CEBM Levels of Evidence. 2009. Available from: www.cebm.net/oxford-centre-evidence-based-medicine-levels-evidence-march-2009/. Accessed 15 July, 2020.

[20] Darling-Hammond L (2006). Constructing 21st-century teacher education. J Teach Educ.

[21] Peterson FL, Cooper RJ, Laird JM (2001). Enhancing teacher health literacy in school health promotion: a vision for the new millennium. J Sch Health.

[22] Bandura A (2004). Health promotion by social cognitive means. Health Educ Behav.

[23] Diener E (2000). Subjective well-being. The science of happiness and a proposal for a national index. Am Psychol.

[24] Masten AS (2001). Ordinary magic. Resilience processes in development. Am Psychol.

[25] Gutman LM, Vorhaus J (2012). The Impact of Pupil Behaviour and Wellbeing on Educational Outcomes.

[26] Zins JE, Bloodworth MR, Weissberg RP, Walberg HJ (2007). The scientific base linking social and emotional learning to school success. J Educ Psychol Consult.

[27] Caemmerer JM, Keith TZ (2015). Longitudinal, reciprocal effects of social skills and achievement from kindergarten to eighth grade. J Sch Psychol.

[28] Sancassiani F, Pintus E, Holte A, Paulus P, Moro MF, Cossu G (2015). Enhancing the emotional and social skills of the youth to promote their wellbeing and positive development: a systematic review of universal school-based randomized controlled trials. Clin Pract Epidemiol Ment Health.

[29] Sklad M, Diekstra R, De Ritter M, Ben J, Gravesteijn C (2012). Effectiveness of school-based universal social, emotional, and behavioral programs: do they enhance students’ development in the area of skill, behavior, and adjustment?. Psychol Sch.

[30] Langford R, Bonell CP, Jones HE, Pouliou T, Murphy SM, Waters E, et al. The WHO Health Promoting School framework for improving the health and well-being of students and their academic achievement. Cochrane Database Syst Rev. 2014(4):CD008958. 10.1002/14651858.CD008958.pub2 PMC11214127 24737131

[31] World Health Organization (WHO). Skills for Health: Skills-Based Health Education Including Life Skills: An Important Component of A Child-Friendly/Health-Promoting School. Geneva: WHO; 2003.

[32] Wilson BM, Pollock PH, Hamann K (2007). Does active learning enhance learner outcomes? evidence from discussion participation in online classes. J Polit Sci Educ.

[33] Pratton J, Hales LW (1986). The effects of active participation on student learning. J Educ Res.

[34] Strayer JF (2012). How learning in an inverted classroom influences cooperation, innovation and task orientation. Learn Environ Res.

[35] Rathmann K, Herke MG, Hurrelmann K, Richter M (2018). Perceived class climate and school-aged children’s life satisfaction: the role of the learning environment in classrooms. PLoS One.

[36] Kaufman Goodstein P. How to Stop Bullying in Classrooms and Schools: Using Social Architecture to Prevent, Lessen, and End Bullying. 1st ed. New York: Routledge; 2013.

[37] Wilson SJ, Lipsey MW (2007). School-based interventions for aggressive and disruptive behavior: update of a meta-analysis. Am J Prev Med.

[38] Weare K, Nind M (2011). Mental health promotion and problem prevention in schools: what does the evidence say?. Health Promot Int.

[39] WHO Constitution. 1948. Available from: https://www.who.int/about/who-we-are/constitution. Accessed 28 May, 2019.

[40] Corin E. The cultural frame: context and meaning in the construction of health. In: Amick III BC, Levine S, Tarlov AR, Walsh DC, eds. Society and Health. New York: Oxford University Press; 1995. p. 272-304.

[41] Eckersley R. Culture, health and well-being. In: Eckersley R, Dixon J, Douglas B, eds. The Social Origins of Health and Well-being. Cambridge: Cambridge University Press. 2001. p. 51-70.

[42] Holmes D (2014). David Napier: cultivating the role of culture in health. Lancet.

[43] Napier AD, Ancarno C, Butler B, Calabrese J, Chater A, Chatterjee H (2014). Culture and health. Lancet.

[44] Grudtsina LY, Filippova AV, Makarova EV, Kondratyuk DL, Usanov VE, Molchanov SV (2017). Preventive pedagogy: methods of research university students’ readiness formation for a healthy lifestyle. Int Electron J Math Educ.

[45] Antonova TV, Kozhanova VV, Kolodovsky AA, SShivrinskaya SE, Kudyashev NK (2016). Health protection features of student youth in research university. Int J Environ Sci Educ.

[46] Seligman MEP, Ernst RM, Gillham J, Reivich K, Linkins M (2009). Positive education: positive psychology and classroom interventions. Oxf Rev Educ.

[47] Breslow L (1999). From disease prevention to health promotion. JAMA.

[48] Centre for Diseases Control (CDC). Prevention, Picture of America. 2010. Available from: https://www.cdc.gov/pictureofamerica/pdfs/picture_of_america_prevention.pdf. Accessed 21 March 2019.

[49] Howard S, Dryden J, Johnson B (1999). Childhood resilience: review and critique of literature. Oxf Rev Educ.

[50] Russo R, Boman P (2007). Primary school teachers’ ability to recognise resilience in their students. Aust Educ Res.

[51] Werner EE. What can we learn about resilience from large-scale longitudinal studies? In: Goldstein S, Brooks RB, eds. Handbook of Resilience in Children. Boston, MA: Springer; 2013. p. 91-105. 10.1007/0-306-48572-9_7

[52] Haggerty RJ, Mrazek PJ. Reducing Risks for Mental Disorders: Frontiers for Preventive Intervention Research. Washington, DC: National Academies Press (US); 1994. 25144015

[53] Patton GC, Sawyer SM, Santelli JS, Ross DA, Afifi R, Allen NB (2016). Our future: a Lancet commission on adolescent health and wellbeing. Lancet.

[54] Walker SP, Wachs TD, Grantham-McGregor S, Black MM, Nelson CA, Huffman SL (2011). Inequality in early childhood: risk and protective factors for early child development. Lancet.

[55] Evans GW, Li D, Whipple SS (2013). Cumulative risk and child development. Psychol Bull.

[56] Elias MJ, Gager P, Leon S (1997). Spreading a warm blanket of prevention over all children: guidelines for selecting substance abuse and related prevention curricula for use in the schools. J Prim Prev.

[57] Mirand AL, Beehler GP, Kuo CL, Mahoney MC (2003). Explaining the de-prioritization of primary prevention: physicians’ perceptions of their role in the delivery of primary care. BMC Public Health.

[58] Michalos AC. Education, happiness and wellbeing. In: Michalos AC, ed. Connecting the Quality of Life Theory to Health, Well-being and Education. Springer, Cham: Springer; 2017.

[59] Annacontini G, Binanti L, Bochicchio F, Celentano MG, Colazzo S, Ellerani P, et al. Istituzioni di Pedagogia e Didattica. Milan: Pearson; 2016.

[60] Pithers RT, Soden R (2000). Critical thinking in education: a review. Educ Res.

[61] Bandura A (2001). Social cognitive theory: an agentic perspective. Annu Rev Psychol.

[62] Chawla L, Cushing DF (2007). Education for strategic environmental behavior. Environ Educ Res.

[63] Becchetti L, Rosati FC (2007). Global social preferences and the demand for socially responsible products: empirical evidence from a pilot study on fair trade consumers. World Econ.

[64] O’Sullivan E. Transformative Learning: Educational Vision for the 21st Century. London: Zed Books; 1999.

[65] World Health Organization. Definition of Health. 1978. Available from: https://8fit.com/lifestyle/the-world-health-organization-definition-of-health/. Accessed 2 April 2019.

[66] Lewallen TC, Hunt H, Potts-Datema W, Zaza S, Giles W (2015). The whole school, whole community, whole child model: a new approach for improving educational attainment and healthy development for students. J Sch Health.

[67] Margiotta U. Teoria della Formazione: Nuovi Orizzonti della Pedagogia. Roma: Carocci; 2015.

[68] Bauman Z. Collateral Damage: Social Inequalities in a Global Age. Cambridge: Polity Press; 2011.

[69] Chen E, Paterson LQ (2006). Neighborhood, family, and subjective socioeconomic status: how do they relate to adolescent health?. Health Psychol.

[70] Currie C, Molcho M, Boyce W, Holstein B, Torsheim T, Richter M (2008). Researching health inequalities in adolescents: the development of the Health Behaviour in School-Aged Children (HBSC) family affluence scale. Soc Sci Med.

[71] Byrne J, Rietdijk W, Pickett K (2018). Teachers as heal/* promoters: factors that influence early career teachers to engage with he h01nd wellbeing education. Teach Teach Educ.

[72] Goleman D. Emotional Intelligence: Why it Can Matter More than IQ. New York: Bantam Books; 1996.

[73] Gardner H. Frames of Mind: The Theory of Multiple Intelligences. New York: Basic Books; 1993.

[74] Tramma S. L’educazione Sociale. Roma : Laterza; 2019.

[75] Nasheeda A, Abdullah HB, Krauss SE, Ahmed NB (2019). A narrative systematic review of life skills education: effectiveness, research gaps and priorities. Int J Adolesc Youth.

[76] Mezirow J (1997). Transformative learning: theory to practice. New Directions for Adult and Continuing Education.

[77] Freinet C. Cooperative Learning and Social Change: Selected Writings of Célestin Freinet. Vol 15. Toronto: James Lorimer & Company; 1990.

[78] Elias MJ, Weissberg RP, Hawkins JD, PerryZins CA, Dodge JE, Kendall KC, et al. The school-based promotion of social competence: Theory, research, practice, and policy. In: Haggerty RJ, Sherrod LR, Garmezy N, Rutter M, eds. Stress, Risk, and Resilience in Children and Adolescents. New York: Cambridge University Press; 1994. p. 268-316.

[79] Lengrand P. Lifelong education: growth of the concept. In: Titmus CJ, ed. Lifelong Education for Adults. Amsterdam: Pergamon; 1989. p. 5-9. 10.1016/B978-0-08-030851-7.50008-4

[80] Banks JA, Au KH, Ball AF, Bell P, Gordon EW, Gutiérrez KD, et al. Learning in and Out of School in Diverse Environments: Life-Long, Life-Wide, Life-Deep. Seattle, WA: Learning in Informal and Formal Environments (LIFE) Center, University of Washington; 2007.

[81] Clunies-Ross P, Little E, Kienhuis M (2008). Self-reported and actual use of proactive and reactive classroom management strategies and their relationship with teacher stress and student behaviour. Educ Psychol.

[82] Sutinen A (2008). Constructivism and education: education as an interpretative transformational process. Stud Philos Educ.

[83] Cribb A, Duncan P (1999). Making a profession of health promotion? Grounds for trust and health promotion ethics. Int J Health Promot Educ.

[84] Strand S. Minority Ethnic Pupils in the Longitudinal Study of Young People in England - DCSF Research Report RR-002. Coventry: University of Warwick, Department for Children, Schools and Families; 2007.

[85] Bradley RH, Corwyn RF (2002). Socioeconomic status and child development. Annu Rev Psychol.

[86] Colao A, Piscitelli P, Pulimeno M, Colazzo S, Miani A, Giannini S (2020). Rethinking the role of the school after COVID-19. Lancet Public Health.

[87] Kjӕrgård B, Land B, Bransholm Pedersen K (2014). Health and sustainability. Health Promot Int.

[88] Morin E. Seven Complex Lessons in Education for the Future. Paris: UNESCO; 2002.

[89] Shepherd J, Pickett K, Dewhirst S, Byrne J, Speller V, Grace M (2016). Initial teacher training to promote health and well-being in schools – a systematic review of effectiveness, barriers and facilitators. Health Educ J.

[90] Jourdan D, Simar C, Deasy C, Carvalho GS, Mannix McNamara P (2016). School health promotion and teacher professional identity. Health Educ.

[91] Nutbeam D (2000). Health literacy as a public health goal: a challenge for contemporary health education and communication strategies into the 21st century. Health Promot Int.

[92] Bonell C, Jamal F, Harden A, Wells H, Parry W, Fletcher A. Systematic review of the effects of schools and school environment interventions on health: evidence mapping and synthesis. Public Health Res 2013;1(1). 10.3310/phr01010 25642578

[93] Poland B, Krupa G, McCall D (2009). Settings for health promotion: an analytic framework to guide intervention design and implementation. Health Promot Pract.

[94] Tjomsland HE, Wold B, Krumsvik RJ, Samdal O. Evaluation research in health promoting schools and related challenges. In: Simovska V, Mannix McNamara P, eds. Schools for Health and Sustainability. Dordrecht: Springer; 2015. p. 365-78. 10.1007/978-94-017-9171-7_17

